# Solitary acrometastasis of the phalanx as initial presentation of an oligometastatic Kirsten rat sarcoma viral oncogene homolog-mutated lung adenocarcinoma: a case report

**DOI:** 10.1186/s13256-023-04127-1

**Published:** 2023-09-18

**Authors:** Alexandra-Ioana Pușcașu, Fabien Moinard-Butot, Delphine Antoni, Roland Schott, Laura Somme

**Affiliations:** 1 Medical Oncology Department, Institut de Cancérologie Strasbourg Europe, 17 Rue Albert Calmette, 67200 Strasbourg, France; 2 Radiation Oncology Department, Institut de Cancérologie Strasbourg Europe, 17 Rue Albert Calmette, 67200 Strasbourg, France

**Keywords:** Acrometastasis, Oligometastatic lung adenocarcinoma, Phalanx metastasis

## Abstract

**Background:**

Acrometastasis is an uncommon finding in non-small cell lung cancer and is usually a sign of multimetastatic disease. Few case reports have suggested solitary digital metastasis as the single secondary lesion of oligometastatic non-small cell lung cancer.

**Case presentation:**

This case report describes an unusual presentation of a Kirsten rat sarcoma viral oncogene homolog-mutated lung adenocarcinoma with a solitary bone metastasis in the fourth finger medial phalanx, which was also the first sign of the disease, in a 63-year-old Caucasian female patient. Digital surgical amputation was performed. After histopathological confirmation and radiological exclusion of other secondary lesions, chemoimmunotherapy in a first-line setting was initiated. A partial metabolic response in the primary lung lesion was observed after four cycles. Maintenance therapy is currently being continued.

**Conclusion:**

Solitary digital metastasis is a rare finding in non-small cell lung cancer. Further studies are needed to investigate the mechanisms behind this particular dissemination process.

## Background

Lung cancer is a leading cause of mortality worldwide, with an estimated 2.2 million new cases and 1.7 million deaths globally in 2020 [1]. Bone metastases (BM) are present in approximately 20–30% of patients with non-small cell lung cancer (NSCLC) at diagnosis and will occur in up to 60% during disease evolution [2, 3]. Most BMs involve the axial skeleton and proximal segments of the limbs [2–5]. Defined as secondary bone lesions situated distally to the elbow or knee, acrometastases (AM) are a rare finding (0.1% of all BM), usually being a sign of a multimetastatic malignancy [6–8]. Physiopathological mechanisms to explain this particular dissemination process remain uncertain [8, 9]. In fewer than 2.5% of lung cancers are BMs the first manifestation of neoplastic disease [10]. Few cases of AM of the hand as the initial disease presentation have been reported [7, 11–15]. Furthermore, oligometastatic NSCLC with only a solitary metastasis at time of diagnosis is rare [16], and only one case with a digital metastasis as a single secondary lesion has been reported [17]. This paper reports a rare presentation of a KRAS-mutated lung adenocarcinoma with a solitary bone metastasis in the fourth finger medial phalanx, which was also the first sign of the disease.

## Case presentation

**Fig. 1:**
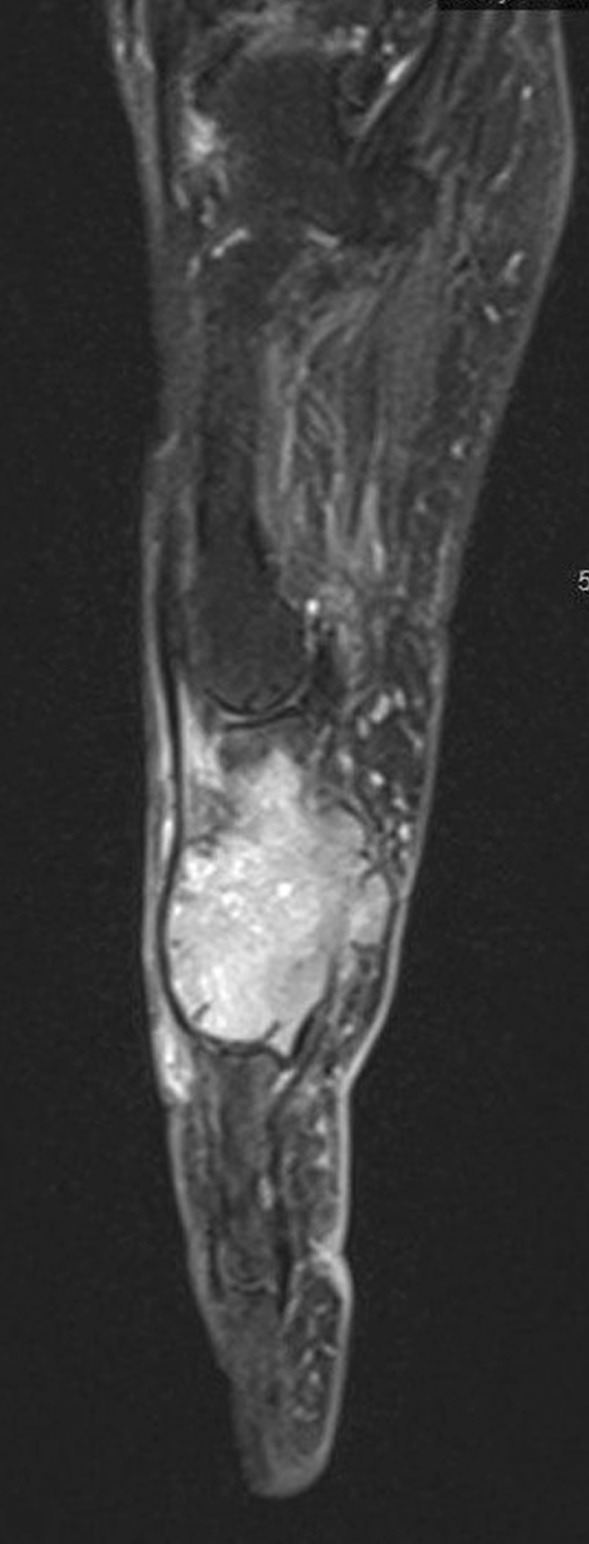
Fourth finger of the left hand, magnetic resonance imaging enhanced sagittal gadolinium T1-weighted section: section osteolytic lesion with the soft tissue component of the left fourth medial phalanx

**Fig. 2:**
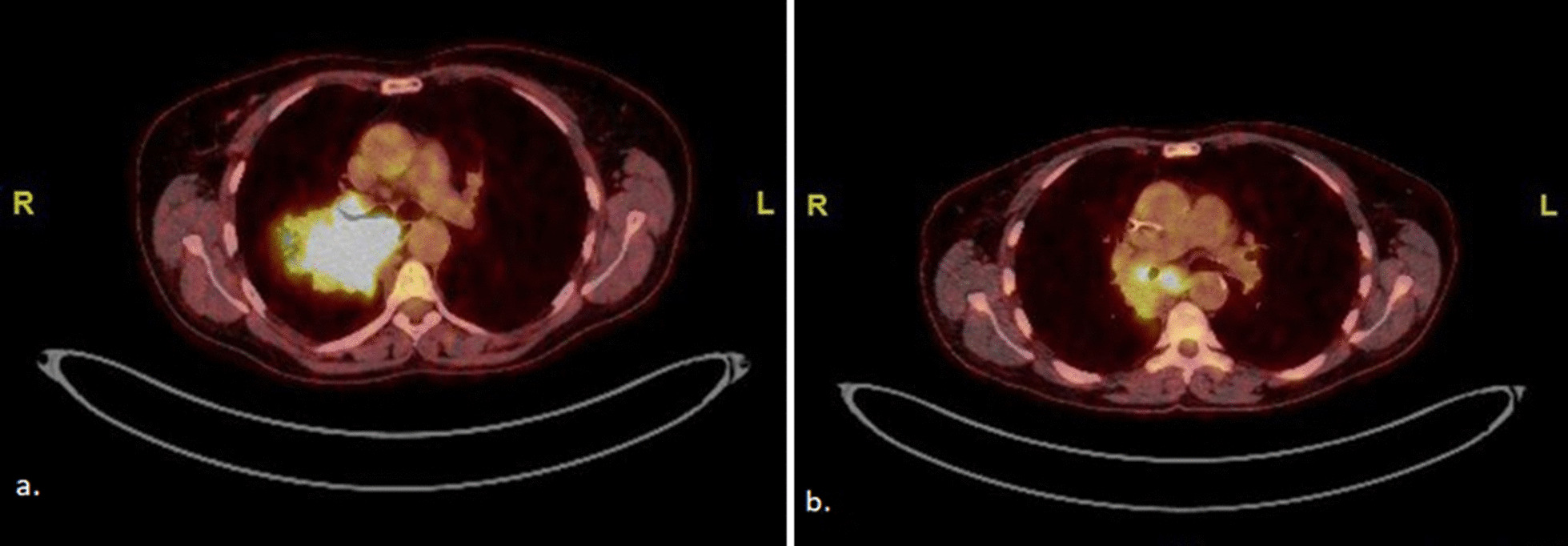
^18^F-FDG PET/CT. **a** Hypermetabolic parahilar primary lung lesion (SUV_max_ 23.9). **b** Partial morphometabolic response (SUV_max_ 8.1) of the primary lesion following four cycles of carboplatin-pemetrexed and pembrolizumab as first-line treatment

A 63-year-old Caucasian woman who was a smoker (15 pack-years) without medical history presented with painful swelling of the fourth left finger, evolving for 1 month, without other symptoms. There was no recent trauma to the left hand. An X-ray of the left hand as well as magnetic resonance imaging (MRI) (Fig. 1) showed a pathological fracture of the medial phalanx of the ring finger as well as a 2-cm osteolytic lesion with a soft tissue component. The differential diagnosis process included other benign entities, such as osteomyelitis, rheumatoid arthritis, gout, or a traumatic lesion. Nevertheless, all laboratory finding were within the normal range (negative autoimmune markers, negative acute phase reactants, no hyperuricemia). A primary osteosarcoma of the bone was suspected, and therefore the amputation of the involved finger was directly performed. Histopathological analysis revealed bone metastasis of a *KRAS* G13D-mutated lung adenocarcinoma. ^18^F-FDG PET/CT showed a 6.8-cm parahilar hypermetabolic primary lesion without any other secondary lesions (Fig. 2a). A cerebral MRI excluded intracranial metastases. Given the presence of acrometastasis, carboplatin, pemetrexed, and pembrolizumab were started as first-line treatment in October 2022. After two cycles of chemoimmunotherapy, ^18^F-FDG PET/CT showed morphological stability of the primary pulmonary lesion associated with a partial metabolic response (SUV_max_ 15.9 versus 23.9). After four cycles of treatment, ^18^F-FDG PET/CT (Fig. 2b) confirmed the partial metabolic response of the primary lung tumor (4.2 cm, SUV_max_ 8.1). She is currently on maintenance therapy with pemetrexed and pembrolizumab. The patient is alive 6 months after her lung adenocarcinoma diagnosis.

## Discussion

This paper reports a rare case of a single acrometastasis of the left fourth phalanx as the initial presentation of an oligometastatic KRAS-mutated NSCLC. Accounting for approximately 0.1% of all bone metastases, acrometastasis remains a rare finding [8]. AMs are more frequently reported in lung cancer and mostly in poorly differentiated carcinomas, but they have also been described in gastrointestinal, genitourinary, and breast malignancies [7–9, 11, 16]. Within the hand, the most common location of acrometastasis appears to be the phalanx, followed by the carpal and metacarpal areas [4, 9].

Digital bone metastasis induces swelling accompanied by insidious-onset pain. Benign diseases are usually first suspected, and the differential diagnosis is made against osteomyelitis, rheumatoid arthritis, gout, or a traumatic lesion [7–9, 11, 18]. Before considering a malignant entity diagnosis, an extended workup should be done, including evaluation of patient history (recent traumatic events, context of gout or other immune diseases), physical examination, review of laboratory data (autoimmune panel, acute phase reactants, uric acid values), or diagnostic imaging [9, 11, 18]. In this reported case, the easy access to imaging allowed an earlier suspicion of an oncological diagnosis.

From the physiopathological perspective, advances in our understanding of the complex pathway of bone metastasis have identified a tropism of lung cancer cells to bones with a higher content of blood marrow and thus higher blood flow [19, 20]. Several factors, such as intrinsic tumor cell properties, appropriate bone marrow environment, and dynamic cell interactions, are thought to be responsible for the dissemination process [2, 3, 20, 21]. Consequently, in NSCLC, more than 80% of bone metastases are in the axial skeleton [2, 3]. Given the scarcity of bone marrow tissue in the bones of the hands, the discovery of secondary lesions in the terminal regions of the extremities is surprising. It is hypothesized that lung cancer cells can more easily make their way to the upper extremities through hematological dissemination, having easier anatomical access to the arterial system. In contrast, data have implied that subdiaphragmatic tumors are more associated with AMs to the owing to the easier spread through the lymphatic system in the lower extremities. Minor repeated traumatic events associated with inflammatory processes and hyperemia may play a role in the pathogenesis of acrometastases [8].

From the molecular biology perspective, no studies have reported a connection between the mutational profile and the ability to develop acrometastases. Approximately 30% of NSCLCs harbor a mutation of KRAS, which mainly occur in codon 12 (90%). A few alterations involve codon 13 [22, 23]. NSCLC with a *KRAS* G12C mutation has the potential to develop more bone metastases compared with controls [24]. However, data about a potential connection between KRAS G13D alterations and metastatic pattern are very scarce.

Overall, AMs are associated with poor prognosis, with 6–8-month overall survival [7–9, 25]. Indeed, AMs are usually a sign of multimetastatic involvement. Therefore, these survival data are inapplicable to our case.

Owing to the rarity of this localization, no studies have reported the benefit of acrometastasis excision in terms of overall survival or quality of life after surgery. A SEER-based study showed a survival benefit for single bone metastasis excision in NSCLC [26]. There is no consensus regarding the best approach for this type of lesion. In our particular case, given the oligometastatic nature of the disease, excision might have improved overall survival.

## Conclusion

Isolated phalanx bone metastasis is an extremely rare occurrence in NSCLC, especially when it is also the first sign of occult disease. The physiopathological mechanisms behind this particular dissemination process remain unclear. More studies are needed to investigate the role of the mutational landscape in acrometastatic development.

## Data Availability

All relevant data are within the manuscript. More clinical information is available from the corresponding author upon reasonable request.

## Abbreviations

AM: Acrometastases
BM: Bone metastases
^18^F-FDG PET/CT: ^18^F-fluorodeoxyglucose positron emission tomography/computed tomography
KRAS: Kirsten rat sarcoma viral oncogene homolog
MRI: Magnetic resonance imaging
NSCLC: Non-small cell lung cancer
SEER: Surveillance, Epidemiology, and End Results
SUV_max_: Maximum standardized uptake value
